# From RNA-seq to large-scale genotyping - genomics resources for rye (*Secale cereale *L.)

**DOI:** 10.1186/1471-2229-11-131

**Published:** 2011-09-28

**Authors:** Grit Haseneyer, Thomas Schmutzer, Michael Seidel, Ruonan Zhou, Martin Mascher, Chris-Carolin Schön, Stefan Taudien, Uwe Scholz, Nils Stein, Klaus FX Mayer, Eva Bauer

**Affiliations:** 1 Plant Breeding, Technische Universität München, Centre of Life and Food Sciences Weihenstephan, 85354 Freising, Germany; 2 Bioinformatics and Information Technology, Leibniz-Institute of Plant Genetics and Crop Plant Research (IPK), D-06466 Gatersleben, Germany; 3 MIPS/IBIS, Institute for Bioinformatics and Systems Biology, Helmholtz Centre Munich, German Research Centre for Environmental Health (GmbH), 85764 Neuherberg, Germany; 4 Genome Diversity, Leibniz Institute of Plant Genetics and Crop Plant Research (IPK), 06466 Gatersleben, Germany; 5 Genome Analysis, Leibniz Institute for Age Research, Fritz-Lipmann-Institute (FLI), 07745 Jena, Germany

**Keywords:** EST resource, next generation sequencing, *Secale cereale *L., Rye5K SNP array, single nucleotide polymorphisms

## Abstract

**Background:**

The improvement of agricultural crops with regard to yield, resistance and environmental adaptation is a perpetual challenge for both breeding and research. Exploration of the genetic potential and implementation of genome-based breeding strategies for efficient rye (*Secale cereale *L.) cultivar improvement have been hampered by the lack of genome sequence information. To overcome this limitation we sequenced the transcriptomes of five winter rye inbred lines using Roche/454 GS FLX technology.

**Results:**

More than 2.5 million reads were assembled into 115,400 contigs representing a comprehensive rye expressed sequence tag (EST) resource. From sequence comparisons 5,234 single nucleotide polymorphisms (SNPs) were identified to develop the Rye5K high-throughput SNP genotyping array. Performance of the Rye5K SNP array was investigated by genotyping 59 rye inbred lines including the five lines used for sequencing, and five barley, three wheat, and two triticale accessions. A balanced distribution of allele frequencies ranging from 0.1 to 0.9 was observed. Residual heterozygosity of the rye inbred lines varied from 4.0 to 20.4% with higher average heterozygosity in the pollen compared to the seed parent pool.

**Conclusions:**

The established sequence and molecular marker resources will improve and promote genetic and genomic research as well as genome-based breeding in rye.

## Background

The improvement of agricultural crops with regard to yield, resistance and environmental adaptation is a perpetual challenge for both breeding and research. With regard to prospected climate changes, improved tolerance against abiotic stresses like drought, low soil fertility, and extreme temperatures is required in crop improvement. The outcrossing species rye shows the highest freezing tolerance among small grain cereals [1] and exhibits excellent tolerance against many biotic and abiotic stresses. Understanding the functional genetic basis of stress tolerance in rye will facilitate the improvement of stress tolerance in wheat (*Triticum aestivum *L.) and barley (*Hordeum vulgare *L.). As a genetic research system, rye is intriguing due to its high genetic variability. In addition to being an economically important crop for Middle and Eastern Europe, rye provides valuable traits for other crops, as a parent of the amphiploid triticale, and as a donor of translocated chromosome segments in wheat [2]. Rye benefits from being diploid and closely related to the more extensively characterized species wheat and barley. While reference sequences of grass genomes have become available for rice [3,4], sorghum [5], *Brachypodium *[6] and maize [7], sequence information for rye is sparse which hampers the exploitation of its genetic potential.

The haploid genome size of rye is more than 8 Gbp [8] which is one of the largest among cereal crops. In addition, 92% of the genome is composed of repetitive sequences [9]. Genetic and genomic resources are limited compared to other *Triticeae*. Currently, 1,073,668 wheat and 501,620 barley ESTs are publicly available whereas only 9,298 rye ESTs are deposited in public databases http://www.ncbi.nlm.nih.gov/dbEST/dbEST_summary.html (release 070111). Publicly available genomic resources for rye are restricted to one BAC library [10], a limited number of genetic markers http://wheat.pw.usda.gov/GG2/index.shtml, and genetic maps with low marker density [11-15].

Next-generation sequencing (NGS) technologies such as Illumina's Genome Analyzer and Roche's 454 sequencing platforms have opened the way to tackle sequencing of large genomes like those of barley and wheat which would be impossible to address by Sanger sequencing [16]. NGS platforms produce hundreds of thousands of sequences in a massively parallel manner, are cost and labour effective and were proven to be reliable and accurate. Several studies have highlighted the success and usefulness of NGS for extending available genomics resources by transcriptome [e.g. [17,18]] and whole-genome [19] sequencing. Furthermore, NGS has been used for gene expression profiling [20], analysis of genome organisation [21], DNA methylation studies [22], and molecular marker development [23], to name few.

Given the large genome size and the lack of sequence information and genomic resources in rye, identification and targeted isolation of genes underlying agronomic traits and understanding of gene function and trait variation is greatly hampered. The aim of the present study was to promote rye genome analysis through massive improvement of the public rye EST resource and development of the first high-throughput SNP genotyping array.

## Methods

### Plant material, RNA and sequencing

Five winter rye inbred lines Lo7, Lo152, Lo225, P87, and P105 were used for cDNA sequencing. Lo7, Lo152, and Lo225 were provided by KWS LOCHOW GMBH (Bergen, Germany) and represent lines from the seed parent and the pollen parent pool of the company's hybrid rye breeding program. P87 and P105 were developed at the Institute of Genetics and Cytology, Minsk, Belarus, and are parents of the mapping population P87 × P105 [24]. Inbred lines Lo7, Lo152, and Lo225 were generated by six selfing generations, whereas P87 and P105 were selfed seven and eight times, respectively. In addition, 54 proprietary inbred lines from the breeding material of KWS LOCHOW GMBH, representing the two breeding pools were investigated. Lines from the pollen parent pool were generated by two to three selfing generations, whereas lines from the seed parent pool have undergone five selfing steps.

To capture a comprehensive part of the rye transcriptome 20 samples of total RNA per inbred line were obtained from a set of plant tissues harvested at five developmental stages and after three stress treatments, respectively (Additional file 1). Three plants per inbred line were pooled to obtain each of the 20 RNA samples. For all non-stress treatments tissue samples from leaves, stems and/or roots were harvested at three- to four-leaf stage, tillering, stem extension, heading and harvest ripe stage. Coleoptiles, florets, early and mature spikes were harvested. To enrich stress induced genes in the cDNA sample, cold stress, dehydration shock, and nutrient-starvation stress treatments were applied in the three- to four-leaf stage. Cold stress was induced by placing plants in a freezer at -15°C. Root, stem and leaf tissues were harvested after 1, 3, and 6 hours of stress treatment and pooled. Dehydration shock experiments were conducted by removing well-watered plants from soil and leaving them on Whatman^® ^3 MM paper (Whatman GmbH, Dassel, Germany) at room temperature [25]. Root, stem, and leaf tissues were harvested after 3, 6, and 12 hours of stress and pooled. Three plants per inbred line were densely planted leading to nutrient-starvation stress. Root and leaf tissues were harvested and pooled. All tissue samples were frozen in liquid nitrogen and stored at -80°C until use. Total RNA was isolated according to manufacturer's instructions using the NucleoSpin RNA Plant kit (#740949, Macherey-Nagel, Düren, Germany) and quantified with the SPECTRONIC GENESYS™ 10 BIO spectrometer (Thermo ELECTRON CORPORATION, Madison, USA).

Five micrograms of the 20 RNA samples of each inbred line were pooled and 100 μg total RNA per inbred line was sent for cDNA synthesis to vertis Biotechnology AG (Freising, Germany). Poly(A)+ RNA was prepared from total RNA. First-strand cDNA synthesis was primed with random hexanucleotide primers. Then 454 sequencing adapters A (5'-GCCTCCCTCGCGCCATCAG-3') and B (5'-CTGAGCGGGCTGGCAAGGC-3') were ligated to the 5' and 3' cDNA ends. Finally, cDNAs were amplified in 20 (Lo152) and 21 (Lo7, Lo225, P87, P105) PCR cycles using a proof reading enzyme. Normalization was carried out by one cycle of denaturation and reassociation of the cDNA. Reassociated ds-cDNA was separated from the ss-cDNA on hydroxylapatite columns to obtain the normalized cDNA samples. After hydroxylapatite chromatography, the ss-cDNA samples were amplified in 8 PCR cycles. The cDNA fraction in the size range of 600 to 800 bp was eluted from preparative agarose gels. As a control, aliquots of the fractionated cDNAs were analyzed on 1.5% agarose gels. Approximately 150 to 250 μg of the normalized, adapter-ligated, and size selected cDNA samples were used for GS FLX 454 sequencing. All 454 sequence raw data were submitted to the EBI sequence read archive (SRA) and are available under the study accession number ERP000274.

### EST resource

#### De novo sequence assembly

**Figure 1:**
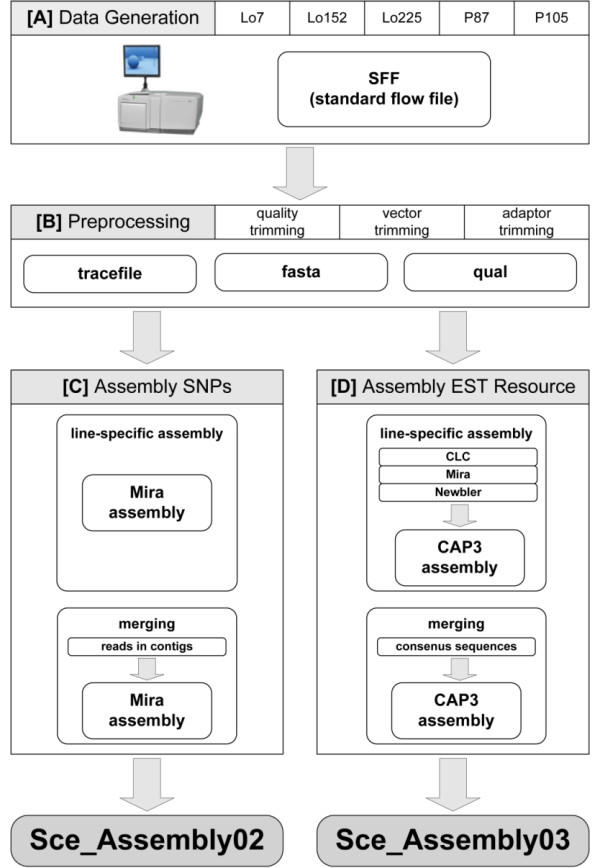
**Pipeline for the assembly procedure of Roche/454 sequence reads**. After data generation [A], sequence (fasta), quality (qual) and trace file information were extracted. Low quality regions, vector and adaptor sequences were removed from raw reads [B]. Preprocessing was finished by subjecting trimmed reads to the line-specific assembly. For establishment of the SNP resource Sce_Assembly02 [C] only reads assembled in contigs of line-specific assemblies were subjected to the merging process of the second assembly using Mira. For establishment of the EST resource Sce_Assembly03 [D] assemblies were computed for each of the five lines separately with CLC assembly cell, Mira, and Newbler and merged by CAP3 assembly. Consensus sequences of all lines were passed to a second CAP3 assembly combining sequences over multiple lines. The resulting sequence set comprises contigs that were confirmed by consensus sequences from two to five lines (multi-line contigs) or contigs that contain reads originating from one line (single-line contigs).

After 454 sequencing, raw sequence reads were passed through quality filtering where cDNA synthesis primer and sequencing adapter sequences were removed. After pre-processing, cleaned and trimmed reads were subjected to inbred line-specific assemblies. Therefore, we adapted the strategy of Kumar and Blaxter [26] for assembling transcriptome data using multiple assembly programs and combining the outcomes to create longer contigs that are less likely to be *in-silico *artefacts brought forth by a single algorithm. The strategy has been modified to be applicable for various lines (Figure 1). We used three independent assemblers to achieve most credible consensus contig sequences. Initially, all reads from each of the five lines were assembled separately into first-order contigs with the programs CLC assembly cell v3.20 http://www.clcbio.com, Mira v3.21 [27] and Newbler v2.5 [28]. While MIRA and Newbler follow the overlap-consensus-layout paradigm (OLC), CLC attempts to find paths in De Bruijn graphs. To obtain line-specific assemblies, all first-order contigs constructed by the three assemblers were merged using the OLC assembler CAP3 [29]. We considered only line-specific contigs whose constituents included first-order contigs from all three assemblers. For EST resource generation (Sce_Assembly03), we employed CAP3 a second time to co-assemble the high confidence line-specific contigs and denoted those supported by constituents from more than one line as multi-line contigs, while contigs with evidence from only one line were deemed single-line contigs. The assembly process of Sce_Assembly03 has been accomplished with a screening for potential DNA and foreign RNA contamination. We applied a BlastN against chloroplast genome sequences of barley (GenBank: NC_008590) and wheat (GenBank: NC_002762), mitochondrial genome sequences of rice (GenBank: AP011077), sorghum (GenBank: DQ984518), and wheat (GenBank: GU985444), and plastids genome sequences of *Brachypodium *(GenBank:EU325680), rice (GenBank: GU592207), sorghum (GenBank: NC_008602), and wheat (GenBank: AB042240). Further purity was gained by excluding hits against CDS sequences of *Acyrthosiphon pisum *(GenBank: ACFK00000000), *Buchnera aphidicola *(GenBank: AE013218), *Fusarium graminearum *(GenBank: AACM00000000), and the draft sequence of *Puccinia triticina *available at the Broad Institute. We discarded contigs from the Sce_Assembly03 sequence set that showed E-values larger than E-20 and the proposed best hits representing at least 10% of the full contig size. The established EST resource Sce_Assembly03 is available from the GABI primary database [30], http://www.gabipd.org.

#### Sequence comparisons

Sequences between the five rye inbred lines potentially differ to a degree that prevents the *de novo *assembly of two lines. Blast [31] comparisons which do not require strict sequence identity were carried out to analyze for overlaps between the different assemblies. Line-specific assemblies generated by CAP3 were used together with the Sce_Assembly03 in an "all versus all" BlastN analysis. Each line-specific assembly as well as the multi-line and single-line contigs of the Sce_Assembly03 were used as both, subject and query sequences. The best query hit to a subject sequence was counted to identify homologs in the respective assemblies. Hits were considered significant when they exceeded a conservative cut-off value of > = 70% identity and 30 bp coverage.

Comparisons of the Sce_Assembly03 against the four currently available protein databases of maize [ZmB73_v5b.60, http://www.maizesequence.org], rice [RAP2, [32]], sorghum [5], and *Brachypodium *[6], two EST databases of barley and wheat (Barley assembly 35 and Wheat assembly WK, http://harvest.ucr.edu), and two full length cDNA (flcDNA) library databases of barley [33] and wheat [34] were performed using BlastX and tBlastX, respectively. Hits were only considered significant when they exceeded a conservative cut-off value of > 70% identity and 30 bp coverage. To prevent hits found based on low-complexity sequences or repeats the Sce_Assembly03 was masked using RepeatMasker [35] and the internal MIPS repeat database [36].

Genome-wide distribution of the Sce_Assembly03 contig sequences was investigated by chromosome-wise BlastX analysis comparing multi-line and single-line contigs with *Brachypodium *protein sequences. Sce_Assembly03 sequences were mapped onto the *Brachypodium *genome by using a sliding window approach with a window size of 0.5 Mb and a shift of 0.1 Mb along the *Brachypodium *chromosomes. The number of BlastX hits and the percent bp coverage of the respective *Brachypodium *genes were determined. These density values were corrected for the number of Ns per window, if the N content exceeded 60% the value was set to zero. Density values were extrapolated to genes [6] or hits (rye) per Mb to facilitate comparisons. To visualize the mapping results heatmaps were created from the density values using the Python matplotlib module in combination with the jet colormap [37].

#### Functional gene annotation

The 115,400 sequences of the Sce_Assembly03 were functionally annotated performing a Blast search with Blast2GO default parameters against the non-redundant (nr) protein sequence database [38] after masking repetitive sequences and excluding the singletons. Gene ontology (GO) terms were assigned using B2G4PIPE http://www.blast2go.org and a locally installed Blast2GO database. The annotation file was extended by its respective GO category - biological process, cellular component, and molecular function - using a custom built Python script that is available upon request.

### SSR mining and SNP discovery

Simple sequence repeat (SSR) motifs within 338,536 contigs of the line-specific assemblies were identified by MISA [39] under standard settings. Out of the five inbred lines, Lo225 was selected as reference dataset as it provided the highest number of SSR containing contigs. The MISA output of the four remaining lines was cross-matched with the Lo225 dataset to detect redundant SSRs. A non-redundant SSR dataset was generated by combining "unique" SSR motifs detected in Lo7, Lo152, Lo225, P87, and P105. Mononucleotide repeat motifs were discarded since monomer runs are known to be the most frequent sequencing errors in Roche/454 data. For experimental validation of *in silico *detected SSRs, primers flanking the SSR motifs were designed using Primer3 [40]. Amplification of the fragments was performed in Lo7, Lo225, P87, and P105 as they are the parents of two mapping populations. Thus, polymorphisms detected between Lo7 and Lo225 and/or P87 and P105 enable the genetic mapping of discovered SSRs. PCR was conducted in a total volume of 20 μl, including 20 ng of genomic DNA, 1× HotStar Taq PCR buffer (Qiagen, Hilden, Germany), 250 nM of each primer, 200 μM dNTPs, and 0.5 U HotStar Taq DNA polymerase (Qiagen, Hilden, Germany). Using a touch-down PCR profile, an initial denaturation step of 15 min at 95°C was followed by 45 cycles of denaturation at 94°C for 1 min, annealing for 1 min, and extension at 72°C for 1 min. Annealing temperature was decreased by 1°C per cycle from 65°C to 55°C and was kept constant for 35 subsequent cycles. A final extension step was performed at 72°C for 10 min. Successful amplification was checked on 1.5% agarose gels.

For the discovery of SNPs in assembled sequences, a second assembly strategy was pursued. Reads assembled in line-specific contigs were selected from all reads and subjected to an overall assembly, merging the extracted reads of all five genotypes (Sce_Assembly02, Figure 1). With this strategy information about nucleotide coverage is maintained which is important for reliable SNP discovery. The Sce_Assembly02 is described in Additional file 2 and is available from the GABI primary database http://www.gabipd.org. The workflow from *in silico *SNP discovery in the Sce_Assembly02 to selection of high confidence SNP candidates was a three-step procedure: First, the tool GigaBayes V0.4.1 [41] was applied with parameter settings given in Additional file 3. Second, characteristics for discovered SNPs were extracted by in-house implementations to compute defined selection criteria for candidate SNPs. Candidate SNPs were filtered by these selection criteria to meet the following requirements: SNPs should be bi-allelic and polymorphic between parents of the two mapping populations Lo7 × Lo225 and/or P87 × P105. For successful probe design they should have a distance to homopolymeres > 5 bp, to the next Indel > 60 bp, and to the contig end > 60 bp. Third, filtered SNPs were manually inspected in the assembled sequences using EagleView [42] to ensure high quality of the SNP genotyping array. We considered putative sequencing errors, SNP position in individual reads, and haplotype information. Oligo-probes for 5,234 SNP were designed and the Rye5K array was produced by Illumina Inc. (San Diego, USA) as Infinium iSelect HD Custom BeadChip. To demonstrate genome-wide coverage of the SNPs represented on the genotyping array SNP containing contig sequences were *in silico *mapped against the *Brachypodium *genome by BlastN analysis.

SNP array performance was assessed by analyzing 59 rye inbred lines including the five inbred lines used for sequencing as well as accessions from barley (Barke, Morex, OWB Dom, OWB Rec, Steptoe), wheat (Chinese Spring, Dream, Mulgara), and triticale (Modus, breeding line SaKa3006). A total of 300 ng genomic DNA per plant was used for genotyping on the Illumina iScan platform and the Infinium HD assay following manufacturer's protocol. The fluorescence images of an array matrix carrying Cy3- and Cy5-labeled beads were generated with the two-channel scanner. Raw hybridization intensity data processing, clustering and genotype calling (AA, AB, BB) were performed using the genotyping module in the GenomeStudio software V2009.1 (Illumina, San Diego, USA). Genotype data were cleaned through exclusion of all SNP assays with more than 5% missing data. Frequencies of the A and B allele for a given SNP were calculated directly by dividing the number of occurrences of one allele (AA + 1/2 AB or BB + 1/2 AB) by twice the number of assayed lines per SNP. Residual heterozygosity of 59 inbred lines was calculated by the relation of heterozygous SNPs (AB) to the number of assayed SNPs per inbred line. Significant deviation of the observed value from the expected value was tested with an exact binomial test using R [43]. Genotyping data of the 10 non-rye accessions were analyzed to investigate the applicability of the Rye5K SNP array to other small grain cereals.

## Results

### Establishment and description of the rye EST resource

#### Assembly

The five independent sequencing runs produced between 364,343 and 681,787 reads corresponding to ~87 and ~166 Mb of raw data per inbred line (Table 1). Subsequent quality filtering and removal of sequencing adapters and cDNA synthesis primers resulted in ~75 to ~145 Mb of high quality sequences per inbred line with median read lengths between 213 and 222 bp. Overall, 2,573,590 high quality reads with a median length of 216 nucleotides were obtained, totalling 548 Mb. The quality filtered reads of the five line-specific cDNA libraries were assembled separately generating between 51,462 and 78,813 contig sequences per line-specific assembly, summing up to 338,536 contigs (Additional file 2). On average each nucleotide in the five line-specific assemblies was covered by 4.5 to 6.2 reads.

**Table 1:** Descriptive statistics of five independent Roche/454 GS FLX sequencing runs.

	Inbred line				
	
	Lo7	Lo152	Lo225	P87	P105
**Raw sequence data**					
Number of sequences	364,343	469,345	572,518	488,829	681,787
Average read length [bp]	239	248	242	240	244
**After quality filtering**					
Number of sequences	363,681	469,208	571,433	488,132	681,136
Average read length [bp]	207	220	213	208	214
Total bp	75,281,967	103,225,760	121,715,229	101,531,456	145,763,104
25% quantile [bp]	203	210	208	203	207
Median [bp]	213	222	218	213	217
75% quantile [bp]	223	236	229	223	228

Consensus sequences created by multiple assembly programs and merged by CAP3 were used to generate the Sce_Assembly03 (Figure 1, Table 2). 89.0% of the reads were assembled into contigs originating from two, three, four, or five inbred lines (multi-line contigs) or from one single inbred line (single-line contigs), respectively. The Sce_Assembly03 resulted in 115,400 sequences including 33,352 multi-line contigs (77.8% of all reads) and 82,048 single-line contigs (11.1% of all reads). 11.0% of all reads failed the quality criteria and were removed from the assembly. The multi-line contig sequence length ranged from 201 bp to 8,636 bp with a L50 length of 1,070 bp. On average, each contig was built from sixty reads in the multi-line contigs and three reads in the single-line contigs.

**Table 2:** Description of the Sce_Assembly03.

	Multi-line contigs	Single-line contigs
**Number of reads**	2,000,855	286,386
**Number of reads/contig**	60	3
**L30 [bp]**	1,527	505
**L50 [bp]**	1,070	333
**L70 [bp]**	727	247
**Number of contigs**	33,352	82,048
< 500 bp	11,188	71,581
501-1000 bp	12,679	8,347
1001-2000 bp	7,693	1,952
2001-5000 bp	1,767	166
> 5000 bp	25	2
**Longest sequence [bp]**	8,636	5,721

#### Sequence comparisons

We compared the five line-specific assemblies generated by CAP3 against each other and against the multi-line and single-line consensus sequences of the Sce_Assembly03 (Table 3). This revealed 52.16% to 78.72% hits between the line-specific assemblies. BlastN analysis of the line-specific assemblies against the multi-line contigs reached up to 87.79% hits. Thus, as expected, a large overlap of represented genes between single-line assemblies can be concluded. However, the remaining 12.21% revealed either pronounced sequence differences (highly polymorphic genes/alleles) or genes that are represented (expressed) in only one of the five rye inbred line samples.

**Table 3:** BlastN comparisons of the five line-specific assemblies generated with CAP3 and the Sce_Assembly03.

	Query						
	Line-specific assembly					Sce_Assembly03	
Subject	Lo7	Lo152	Lo225	P87	P105	Multi-line contigs	Single-line contigs
**Line-specific assembly**							
Lo7		52.2	56.1	61.8	56.9	76.1	35.5
Lo152	67.7		54.3	59.6	56.0	77.1	49.5
Lo225	77.6	58.3		68.7	63.8	84.2	53.5
P87	74.4	55.4	59.9		60.9	82.8	40.6
P105	78.7	59.5	63.8	70.2		87.8	47.5
**Sce_Assembly03**							
Multi-line contigs	85.2	64.4	69.6	78.0	72.3		35.3
Single-line contigs	59.1	64.4	67.3	59.2	62.4	58.5	

Values show percent hits of query sequences counting the first best hit in each comparison.

**Figure 2:**
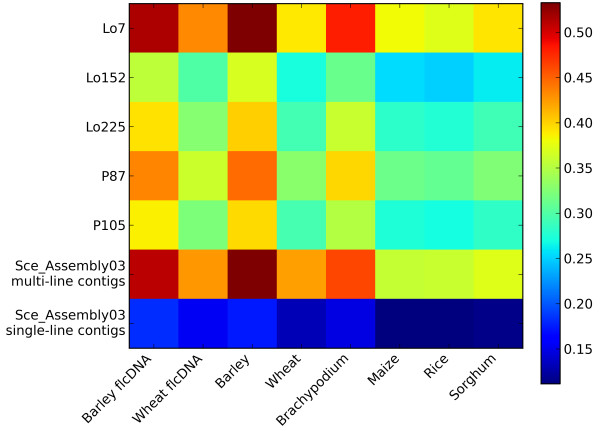
**Heatmap of (t)BlastX analysis results to public model grass genomes and *Triticeae *EST and full length cDNA (flcDNA) resources**. Contig sequences from the line-specific assemblies generated by CAP3 and the Sce_Assembly03 were aligned to public barley and wheat EST and flcDNA sequences and to *Brachypodium*, maize, rice, and sorghum genomic sequences. Percent hits to individual databases were counted using a 70% similarity cutoff and visualized in colours (colour code shown on the right).

The sequence homology between the line-specific assemblies and the Sce_Assembly03 with the reference genomes of *Brachypodium*, maize, rice, and sorghum, and available flcDNA and EST collections from wheat and barley, respectively, was investigated by (t)BlastX comparisons (Figure 2). Most homologs were identified in comparison to barley sequences, followed by *Brachypodium*, wheat, sorghum, maize and rice. Contig sequences of the line-specific assemblies and multi-line contigs of the Sce_Assembly03 showed a high homology to the public sequence databases. Low homology was detected for the single-line contigs of the Sce_Assembly03. This finding can be attributed to the sequence length which is about two thirds shorter than that of multi-line contigs (Table 2). Multi-line contigs of the Sce_Assembly03 yielded more than 65% hits with either barley or wheat flcDNA and HarvEST assemblies (data not shown). Through tBlastX comparisons of the Sce_Assembly03 against the genome sequences of *Brachypodium*, maize, sorghum, and rice we were able to tag fragments from about 46.3%, 35.9%, 37.2% and 36.2% of the reference gene repertoires. From 33,352 multi-line and 82,048 single-line contigs 22,926 (68.7%) and 23,406 (28.5%) revealed a hit to at least one of the public grass sequence resources. The genes comprised in the rye cDNA libraries indicated no bias for or against a certain region of the rye genome when comparing the Sce_Assembly03 contig sequences to the *Brachypodium *genome (Additional file 4). The dense gene content in the distal regions of the *Brachypodium *chromosomes as well as the gene poor regions around the centromeres were well covered by Sce_Assembly03 contig sequences.

#### Functional gene annotation

After masking repetitive sequences of the Sce_Assembly03 111,150 sequences (32,725 multi-line and 78,425 single-line contigs) remained for Blast2GO analysis. Out of these sequences 49,294 revealed a hit against the nr database and subsequently 35,356 (71.7%) unique rye contig sequences (16,970 multi-line and 18,386 single-line contigs) were assigned to one or more GO annotations. In total 35,186, 38,280 and 51,950 GO terms were obtained for biological processes, cellular components and molecular functions, respectively (Additional file 5). Across the three GO categories, 4,997 unique GO terms were identified. More than 350 sequences in the Sce_Assembly03 were related to biotic and abiotic stress response (data not shown).

### Marker discovery, SNP array design and high-throughput genotyping

#### SSR marker development

Within the 338,536 contigs of the line-specific assemblies a fraction of 12,317 (3.6%) contigs contained SSR motifs. Primer sequences could be designed for 5,230 of these contigs. Restriction to di-, tri-, tetra-, penta- or hexa-nucleotide motifs reduced the number of SSR candidates to 3,799. Cross-match analysis filtered a final SSR dataset comprising 1,385 unique, non-redundant SSRs (Additional file 6). A random subset of 155 SSRs was chosen for experimental validation by PCR amplification of the four parental genotypes Lo7, Lo225, P87, and P105. 146 primer pairs (94%) immediately amplified fragments of expected size without further optimization of PCR conditions. Twelve primer combinations produced fragments larger than expected indicating the presence of introns. These were excluded from further analyses. Finally, 61 (46%) out of 134 PCR products with expected fragment size revealed naked-eye polymorphisms on agarose gels between either P87 and P105 (29) or Lo7 and Lo225 (37).

#### SNP discovery

SNP discovery requires sufficient coverage with high quality sequence reads in order to allow for distinguishing true SNPs from sequencing errors. Therefore, the assembly Sce_Assembly02 was performed that excluded singletons from the line-specific assemblies when merging sequences of the five inbred lines. Overall 277,033 putative polymorphisms in 138,339 contigs cumulating 55 Mb consensus sequences were identified in a first data mining step using GigaBayes. The number of SNP candidates was reduced to 17,917 by filtering those SNPs that fulfilled the selection criteria and quality requirements such as bi-allelic and polymorphic between parents of the two mapping populations Lo7 × Lo225 and/or P87 × P105, distance to homopolymeres > 5 bp, distance to the next Indel > 60 bp, and distance to the contig end > 60 bp. Subsequent manual inspection in the Sce_Assembly02 reduced the dataset to 5,211 SNP candidates from 3,961 contigs. This dataset together with additional 23 SNPs discovered in non-public rye sequences was used for the design and production of the Rye5K SNP genotyping array. Out of the 3,961 unique contigs, 2,835 contigs (71.6%) were *in silico *mapped to the *Brachypodium *genome. The contigs were evenly distributed with 826, 641, 688, 416, and 262 hits on chromosomes Bd1 to 5, respectively (Additional file 4). Blast2GO analysis of 3,961 contig sequences represented on the Rye5K array assigned 2,096 sequences with associated GO identifiers (Additional file 7).

#### Application of the Rye5K SNP array

**Figure 3:**
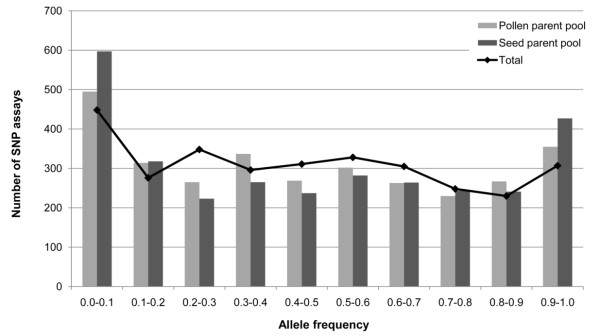
**Distribution of allele frequencies for evaluable SNPs on the Rye5K SNP array**. Allele frequencies observed in total and separately in the rye breeding seed parent and pollen parent pools belong to one category if the value is > the left category border and ≤ the right category border. Allele frequency values equal to 0 and 1 fall into the first and last category, respectively.

The performance of the Rye5K SNP array was tested on the five inbred lines selected for RNA-*seq*, 54 additional rye inbred lines, and 10 non-rye accessions. Out of the 5,234 SNPs, 4,557 (87%) generated signals and between 2,970 (57%) and 3,148 (60%) were successfully called for the 59 rye inbred lines representing the hybrid rye seed parent and pollen parent pools (Table 4 Additional file 8). Based on genotyping results for the five inbred lines used for SNP discovery, 3% of the *in silico *detected SNPs turned out to be false positives. Allele frequencies in rye were evenly distributed from 0.1 to 0.9 (Figure 3). A small proportion of 12.3% called SNPs turned out to be monomorphic in the independent set of 54 inbred lines not used for SNP discovery with slightly increasing values when looking separately at the pollen parent (15.7%) and the seed parent (13.7%) pools.

**Table 4:** Heterozygosity of five sequenced rye inbred lines after genotyping with the Rye5K array.

	Inbred line				
	
	Lo7	Lo152	Lo225	P87	P105
Loci total	3,145	3,133	3,134	3,148	3,127
Homozygous loci	3,004	3,005	2,987	2,997	2,988
Heterozygous loci	141	128	147	151	139
Generation	F_7_	F_7_	F_7_	F_7:10_	F_6:9_
Expected heterozygosity [%]	1.6	1.6	1.6	1.6	3.1
Observed heterozygosity [%]	4.5***	4.1***	4.7***	4.8***	4.4*

Significant (***: *p*-value < 0.01, *: *p*-value < 0.05) deviation from the expected level of heterozygosity is indicated.

Genotyping data were used to calculate the observed residual heterozygosity of the rye inbred lines. The observed percentage of heterozygous loci for each line varied between 4.1 and 4.8% in the five rye inbred lines used for 454 sequencing and between 4.0 to 20.4% in the 54 inbred lines from the two heterotic breeding pools. On average, a higher level of residual heterozygosity was observed for the pollen parent pool (11.5%) than for the seed parent pool (5.5%).

Applicability of the Rye5K SNP array to other small grain cereals was investigated. Out of the 4,557 SNP assays that generated a signal in rye, 63.0% (2,871), 75.8% (3,452), and 84.1% (3,831) could be scored in barley, wheat, and triticale, respectively. However, 86.7, 91.6, and 76.5% of the scored SNPs did not show a polymorphism between the investigated barley, wheat, and triticale accessions.

## Discussion

### Dual-purpose transcriptome sequencing

In this study we report the establishment of rye genomic resources comprising 115,400 EST sequences, 1,385 SSRs, more than 5,000 SNPs, and the Rye5K SNP array for large-scale genotyping. NGS was used to generate transcriptome sequences of the five rye inbred lines Lo7, Lo152, Lo225, P87, and P105. The number of reads per sequencing run of the present study was in line or even surpassed results obtained in other studies [17,23,44]. Due to the massive number of 2.5 Mio read sequences obtained by 454 sequencing the *de novo *assembly of such datasets remains a computational and bioinformatic challenge. Two purpose-oriented assembly strategies were followed in order to first provide a comprehensive EST resource and second enable discovery of polymorphisms between inbred lines. A second assembly on top of the five line-specific assemblies reduced the possibility of creating chimeric artefacts in the Sce_Assembly03. In addition, sequence redundancy introduced by variations between lines is removed. This was achieved by bringing together related sequences while accepting line specific nucleotide differences. In contrast this fact was essential for SNP detection, where only reads that were pre-assembled in line-specific contigs were subjected to the Sce_Assembly02. Thus, information about allele coverage at the SNP position was retained which increased the reliability of SNP candidates. A challenge in our study was the detection of SNPs without a reference sequence. Many SNP detection tools such as GMAP [45] or MAQ [46] are only applicable to *de novo *assemblies that are aligned to a reference sequence. This was a strong challenge in our approach and much effort was invested in the detection of high confidence SNPs. Manual inspection of SNP candidates in more than 10,000 contigs indicated that many sequencing errors occurred in the beginning of read sequences which, as a consequence, lead to false positives. Exclusion of SNP candidates detected in such regions of read sequences might reduce the false positive rate and improve automated tools that detect polymorphisms in *de novo *assembled sequence data without a reference sequence.

Genome sequencing has progressed rapidly in model plants. Given the increased sequencing throughput and the decreasing costs, NGS technologies pave the way for sequencing even large genomes [47-49]. Although of major importance for research and breeding, sequence resources for rye were sparse imposing serious limitations for trait mapping, association studies, and functional genomics in rye. Rye is of interest especially for Middle and Eastern European economic markets due to its high tolerance to abiotic stresses. As a first step towards deciphering the rye genome we aimed to sequence a large portion of the rye transcriptome. To achieve this we first sampled RNA from plants under various stress conditions, different plant tissues and developmental stages. Rye-specific sequences e.g. related to stress tolerance were generated in the present study which are indispensable for functional genomic studies in rye. Second, we reduced the complexity of the transcriptome by cDNA normalization prior to sequencing. cDNA normalization lead to a significant increase in transcriptome sequencing efficiency by equalizing the representation of high, medium and rarely expressed transcripts in the cDNA population [50-52]. Since many transcripts are temporally and/or spatially expressed during plant development, RNA pooled from different tissues at different developmental stages ensured the coverage of temporal- and spatial-specific transcripts.

### Linking rye to grass genome sequence resources

To assess, how much of the rye transcriptome is represented by the established EST resource, we compared the Sce_Assembly03 sequences to currently available grass genome, flcDNA, and EST sequences. Generally, the number of sequences with significant BlastX hit in public databases was higher for multi-line contigs than for single-line contigs. This finding is in line with results of Schafleitner et al. [53] who compared EST sequences of sweet potato (*Ipomea batatas*) with sequences contained in the UniRef100 protein database.

The overall gene content across the grass subfamilies *Ehrhartoideae *(rice), *Panicoideae *(maize, sorghum), and *Pooideae *[6] is in a similar range. A total of 25,532 protein coding gene loci were found for *Brachypodium *[6] which is in line with rice [RAP2, 28,236 protein coding gene loci, [32]], maize [ZmB73_v5b.60, 39,656 protein coding loci, [7]], and sorghum [v1.4, 27,640 protein coding gene loci, [5]]. Due to a close evolutionary relationship with these model genomes a pronounced overlap with rye transcripts was expected. The comparison of the Sce_Assembly03 against flcDNA, EST, and genomic sequences revealed a higher homology to barley, *Brachypodium*, and wheat than to maize, rice, and sorghum which was expected, as rye is phylogenetically more closely related to other members of the *Pooideae *than to maize, rice, and sorghum [54,55]. The GO annotation analysis reveals that a broad spectrum of genes was sampled in our normalized cDNA pool from multiple tissues and developmental stages. The large number of reads generated by 454 sequencing entails a substantial gain at the level of gene discovery which provides a valuable resource for forward and reverse genetics approaches in rye as well as for comparative gene analyses. A significant fraction of multi-line contigs (31%) gave no hits with the public grass sequence resources. In part this finding can be attributed to species specific and tribe specific genes and gene families. The *Pooideae *contain 265 subfamily-specific gene families leading to subfamily-specific Blast hits [6]. Given our stringent BlastX/tBlastX cut-off value of > 70% sequence identity, non-conserved and non-coding sequences such as 3'- or 5'- untranslated regions and non-coding RNAs are assumed to contribute to the fraction that lacks homology with other grass species. Around 2% of all rye 454 reads revealed hits to the MIPS Repeat Element database [36], suggesting that transcriptional activity of retrotransposons contributed to the sampled RNA pool. Transcriptome sequencing in two rice subspecies detected alternative splicing patterns in about half of the rice genes and more than 15,000 novel transcriptional active regions of which more than half had no homolog in public protein data [56]. This might suggest that the rye EST resource contains rare, tissue-specific and/or stress-related transcripts that are not represented in sequence resources of the closely related species wheat and barley despite their extensive EST resources. It is anticipated that rye transcriptome sequence analysis will greatly benefit from a reference genome sequence for a member of the *Triticeae *family. Whole genome sequencing is in progress for barley [49,57] and wheat [58] and exploratory BAC end sequencing of rye 1RS-specific BAC libraries [59] has been reported. *In silico *mapping of rye ESTs to the model genome of *Brachypodium *revealed an even distribution of rye transcripts when anchored to their *Brachypodium *homologs. The large extent of synteny between grass genomes will facilitate the construction of a virtual gene map of rye representing the ancestral gene scaffold. Genetic mapping of the SNPs represented on the Rye5K array and of SSRs developed from our rye ESTs is underway and will lead to fine-scale comparative maps between rye and other grasses. A fully annotated genome sequence for rye is still out of reach due to the complexity and highly repetitive nature of the rye genome. However, with the tools established in our study, rye catches up with other grass genome resources and a far more detailed glimpse into the rye genome and its evolution will be possible.

### Molecular toolbox for rye

Sequence information of the five rye inbred lines was used to detect sequence variation that was transferred into more than 1,300 SSRs and about 5,000 SNPs. Molecular markers have been developed for a range of crop species and play an essential role in modern plant breeding. They have been used to monitor DNA sequence diversity within and among species, to identify genes responsible for desired traits, to disclose sources of genetic variation that allow for the production of new varieties by introducing favorable traits from landraces and related grass species, and to manage backcrossing programs [60]. Together with amplified fragment length polymorphisms (AFLPs), SSRs are currently the most popular marker system in cereals. They have been developed for major crop plants including cereals and when applied in breeding programs this marker system is predicted to lead to accelerated progress [61]. Currently, the availability of public rye SSRs is very limited. Our resource significantly increases this marker resource that might facilitate the assessment of genetic variability and the estimation of genetic distances between populations. Besides SSRs the marker system receiving the greatest attention nowadays are SNPs [62]. SNPs have shown huge potential in highly efficient fingerprinting, genetic map construction, marker assisted selection as well as population and evolutionary genetics. The Rye5K SNP array provides a powerful new resource for large-scale genotyping in molecular and genome-centric research in rye. Recently whole-genome genotyping arrays became available for crops and livestock and are used for genome-wide association studies and to investigate genetic variation [e.g. [63]]. In a pilot experiment, we analyzed 59 rye inbred lines including the five lines used for sequencing with the Rye5K SNP array to estimate the degree of residual heterozygosity. Theoretical expectation after two, three or six cycles of selfing is about 12.5%, 6.3%, and 1.6%, respectively. Genotyping of these 59 lines using the Rye5K array showed that the degree of heterozygosity significantly (*p*-value < 0.05) exceeds this theoretical expectation. This might be in part explained by the allogamous behaviour of rye resulting in remaining heterozygosity [64]. Despite forced selfing during inbred line production some degree of cross-pollination cannot be excluded as the seed was produced as single-ear progenies in a commercial breeding program. The lower levels of residual heterozygosity observed for the seed parent pool is in agreement with the higher advanced selfing generations in rye seed parent lines (P. Wilde, personal communication). A detailed analysis of sequences that remained heterozygous indicated sequences belonging to large gene families, such as transferases and hydroxylases. Detection of SNPs in paralogs or members of gene families may mimic a substantial part of the detected heterozygosity, thus leading to an overestimation of the true remaining heterozygosity in the rye inbred lines.

## Conclusions

In conclusion, the Sce_Assembly03 provides a new and comprehensive EST resource that integrates rye in the comparative analysis between small grain cereals. The Rye5K SNP array allows the analysis of large sets of individuals to obtain genotyping data for association studies, estimating linkage disequilibrium, and population genetic approaches. Our genomic resources comprise 115,400 EST sequences, 1,385 SSRs, more than 5,000 SNPs, and the Rye5K SNP array for large-scale genotyping that will improve and promote genetic and genomic research as well as genome-based breeding in rye.

## Authors' contributions

GH prepared the sequencing samples, participated in the bioinformatic analyses, conducted the genotyping, and evaluated the genotyping data. TS, MM, and US carried out the processing and assembly of 454 reads and gave the descriptive statistics for them. KFXM and MS performed the BLAST analyses, functional annotations, and sequence comparisons along the *Brachypodium *chromosomes. NS and RZ developed and examined the SSR markers. EB, GH, and TS developed the Rye5k SNP array. CCS, EB, KFXM, NS, and US designed the study. EB, GH, MS, RZ, and TS drafted the manuscript. All authors read and approved the final manuscript.

## Supplementary Material

Additional file 1 **Set of plant tissues for RNA extraction**. RNA of each rye inbred line was extracted from plant tissues exposed to various stress treatments and harvested at different developmental stages. Click here for file

Additional file 2 **Establishment and description of the Sce_Assembly02 generated for *in silico *SNP mining**. The Sce_Assembly02 was performed in three steps using the MIRA assembler V2.9 on integrated standard settings: Firstly, raw sequence reads surpassed a quality filtering process where 454 sequencing adapter and cDNA synthesis primer sequences as well as low quality reads were removed. Secondly, the cleaned and trimmed sequence reads were subjected to a line-specific assembly where reads of each inbred line were aligned in a separate assembly run. Non-aligned reads in the line-specific assemblies, i.e. singletons, were rejected. Thirdly, those reads that merged into contigs in the line-specific assemblies were moved further to the Sce_Assembly02 starting again with the cleaned and trimmed reads, but now from all five inbred lines. This strategy resulted in contig sequences that were used for SNP detection and subsequently for the design of the high-throughput genotyping SNP array. With regard to SNP discovery this assembly allowed the deduction of critical information about the SNP position like allele coverage. Click here for file

Additional file 3 **GigaBayes parameters**. Only parameters different from GigaBayes program default settings are listed. Click here for file

Additional file 4 **Association of multi-line and single-line contigs of the Sce_Assembly03 to the *Brachypodium *chromosomes Bd1 to Bd5**. The four heatmaps per chromosome are depicting the density of *Brachypodium *genes, homologous rye sequences, contigs represented on the Rye5k SNP array, and SNPs that were heterozygous among 59 rye inbred lines (from top to bottom) by going along the *Brachypodium *chromosomes in a sliding window with 0.5 Mb window size and a 0.1 Mb shift and determining for each window the number and percent bp coverage of the respective tagged genes. The density values were corrected for the number of Ns per window, if the N content exceeded 60% the value was set to zero and drawn in white color. The number was extrapolated to number per Mb to facilitate comparisons. The heatmaps were created from density values using the Python pylab module in combination with the jet colormap (low to high values from blue to red). Minimum, maximum, and mean number of genes/Mb in *Brachypodium *and hits/Mb in rye, respectively, were given on the left of each map. The ruler on top gives the chromosome length in Mb. Click here for file

Additional file 5 **GO categories found in the Sce_Assembly03 multi-line and single-line contig sequences on Blast2GO level 2**. Categories with an occurrence less than 0.05% were summarized in "others". Click here for file

Additional file 6 **SSR motifs detected in 338,536 contigs of the five line-specific assemblies**. Mononucleotide repeat motifs were discarded. Mixed motifs describe two close SSR motifs which are separated by less than 100 bp. Click here for file

Additional file 7 **Description of the Rye5K SNP array**. SNP containing contigs represented on the Rye5K SNP array were listed including candidate SNP position, probe design sequences provided to Illumina Inc. (San Diego, USA), and GO annotations. Click here for file

Additional file 8 **Observed residual heterozygosity of 54 rye inbred lines representing the two heterotic pools. Heterozygosity was calculated based on genotyping data obtained with the Rye5K SNP array**. Lines from the pollen parent pool were in generations F_3 _to F_4_, lines from the seed parent pool were in generation F_6_. Click here for file
