# Supplementary figures and images for: From RNA-seq to large-scale genotyping - genomics resources for rye (Secale cereale L.)
Source: BMC Plant Biol. 2011 Sep 28;11:131. doi: 10.1186/1471-2229-11-131 (PMC3191334; doi:10.1186/1471-2229-11-131)

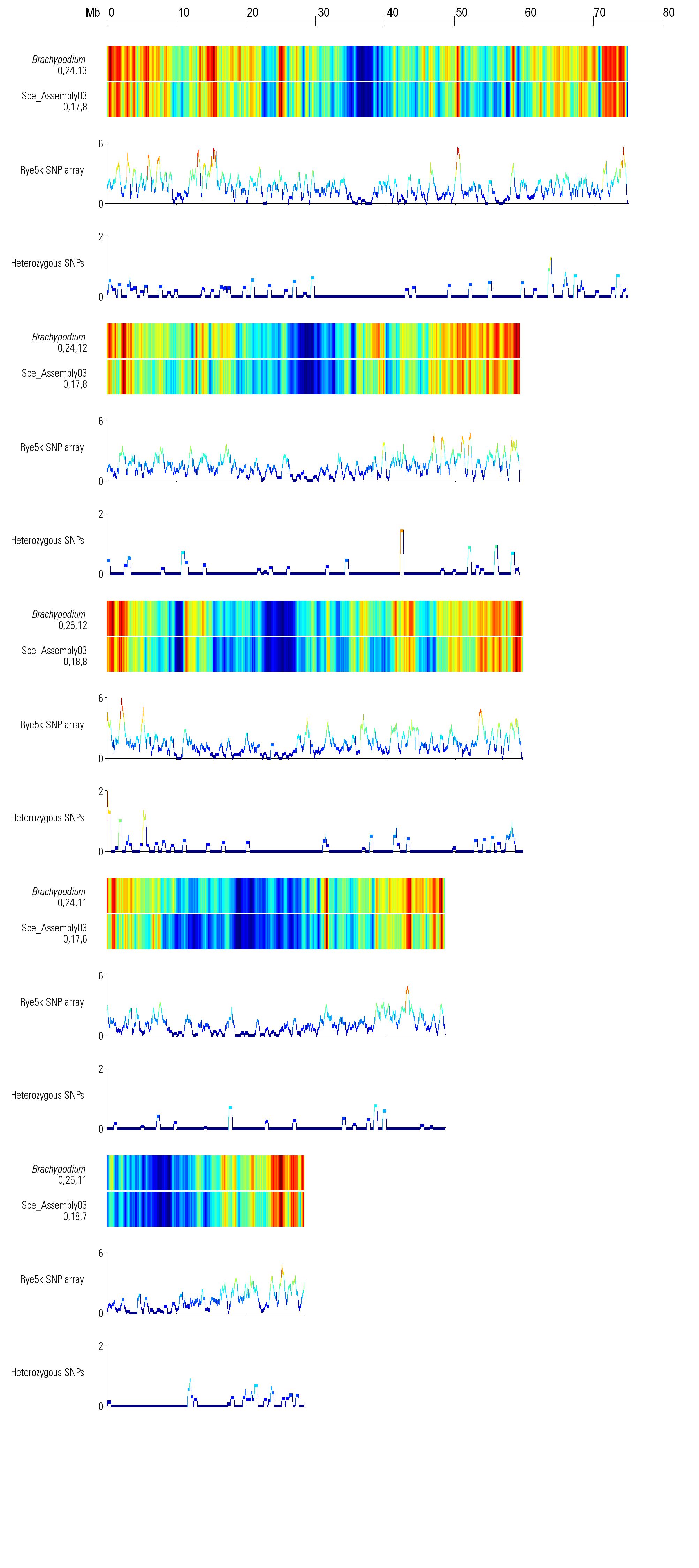

Supplement: Additional file 4 — Association of multi-line and single-line contigs of the Sce_Assembly03 to the Brachypodium chromosomes Bd1 to Bd5. The four heatmaps per chromosome are depicting the density of Brachypodium genes, homologous rye sequences, contigs represented on the Rye5k SNP array, and SNPs that were heterozygous among 59 rye inbred lines (from top to bottom) by going along the Brachypodium chromosomes in a sliding window with 0.5 Mb window size and a 0.1 Mb shift and determining for each window the number and percent bp coverage of the respective tagged genes. The density values were corrected for the number of Ns per window, if the N content exceeded 60% the value was set to zero and drawn in white color. The number was extrapolated to number per Mb to facilitate comparisons. The heatmaps were created from density values using the Python pylab module in combination with the jet colormap (low to high values from blue to red). Minimum, maximum, and mean number of genes/Mb in Brachypodium and hits/Mb in rye, respectively, were given on the left of each map. The ruler on top gives the chromosome length in Mb. [file 1471-2229-11-131-S4.PNG]

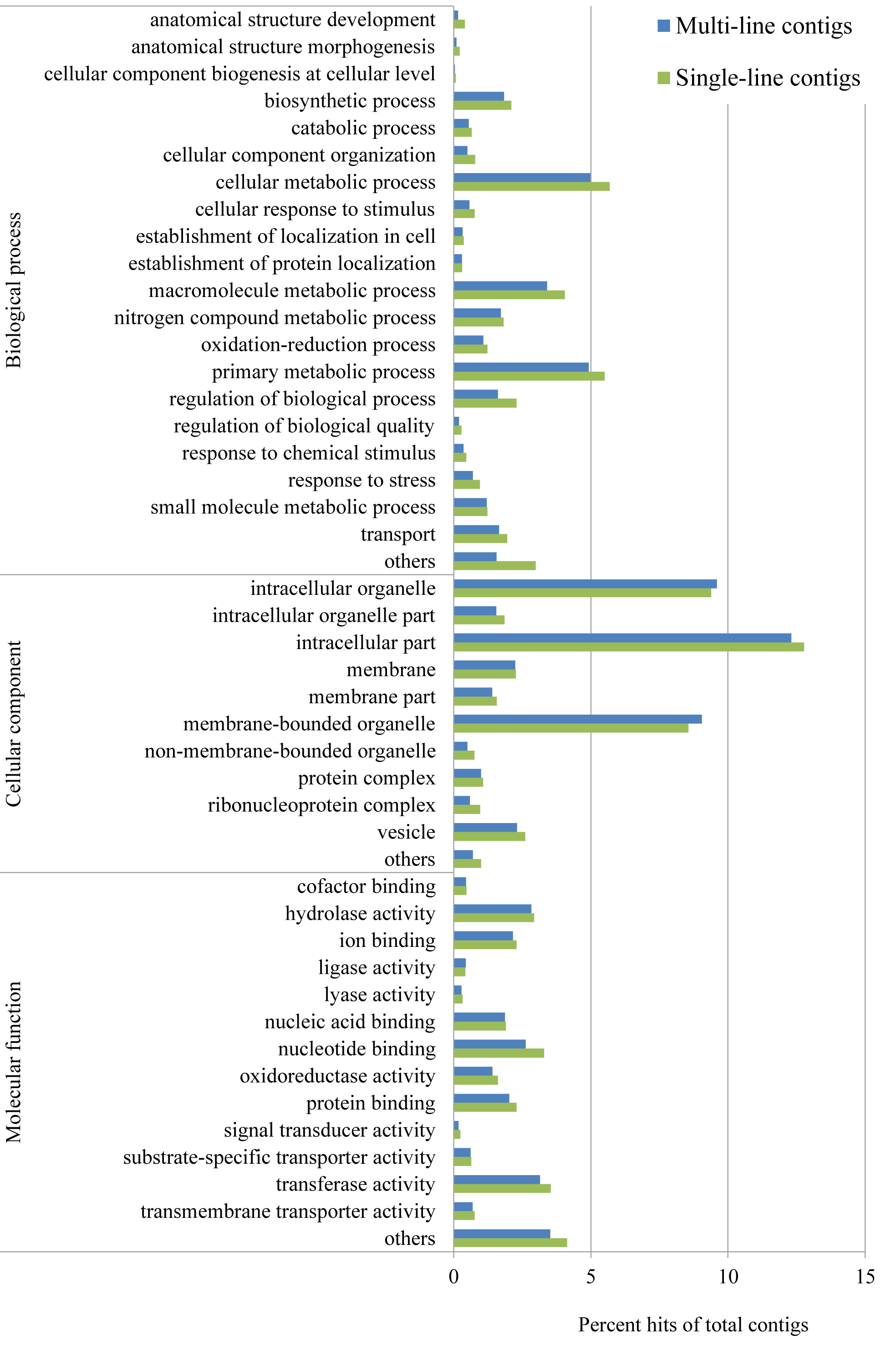

Supplement: Additional file 5 — GO categories found in the Sce_Assembly03 multi-line and single-line contig sequences on Blast2GO level 2. Categories with an occurrence less than 0.05% were summarized in "others". [file 1471-2229-11-131-S5.PNG]
